# Apart but not Alone? A cross-sectional study of neighbour support in a major UK urban area during the COVID-19 lockdown

**DOI:** 10.35241/emeraldopenres.13731.1

**Published:** 2020-06-10

**Authors:** Mat Jones, Amy Beardmore, Michele Biddle, Andy Gibson, Sanda Umar Ismail, Stuart McClean, Jo White

**Affiliations:** 1 Health and Applied Social Sciences, University of the West of England Bristol, Bristol, BS161QY, UK; 2 National Institute for Health Research, Applied Research Collaboration West (ARC West), Bristol, BS1 2NT, UK

**Keywords:** Community activism, mutual aid, informal care, health inequalities, social capital, crisis response and recovery, voluntarism

## Abstract

**Background: **Evidence from a range of major public health incidents shows that neighbour-based action can have a critical role in emergency response, assistance and recovery. However, there is little research to date on neighbour-based action during the 2020 coronavirus pandemic. This article reports on a survey of people engaged in supporting their neighbours in weeks three and four of the UK COVID-19 lockdown.

**Methods: **Members of area-based and community of interest COVID-19 support groups in the Bristol conurbation were invited to complete an online survey. Of 1,255 people who clicked on the survey link, 862 responded; of these, 539 responses were eligible for analysis.

**Results: **Respondents reported providing a wide range of support that went beyond health information, food and medical prescription assistance, to include raising morale through humour, creativity and acts of kindness and solidarity. A substantial proportion felt that they had become more involved in neighbourhood life following the lockdown and had an interest in becoming more involved in future. Neighbour support spanned all adult age groups, including older people categorised as being at-risk to the virus. With respect to most measures, there were no differences in the characteristics of support between respondents in areas of higher and lower deprivation. However, respondents from more deprived areas were more likely to state that they were involved in supporting certain vulnerable groups.

**Conclusions: **As with previous research on major social upheavals, our findings suggest that responses to the viral pandemic and associated social restrictions may increase existing social and health inequalities, and further research should explore this issue in more depth.

## Introduction

At times of public health emergency, neighbours can play a critical role in responding to the needs of people who are adversely affected, vulnerable or at-risk. Neighbour action can also assist the wider interests of communities more generally. Globally, government-directed lockdowns in response to the coronavirus pandemic of 2020 appear to have stimulated a widespread rise in social action at the local level of neighbourhoods. The aim of this article is to characterise some central features of the support arising at the neighbourhood level. This is in view of the current changes that are reshaping neighbourhood life towards greater social fluidity and digital mediation, despite persistent social divisions.

Neighbour support is an important feature of everyday life that can contribute towards the health and wellbeing of a range of social groups ( Cramm *et al*., 2013; and Vyncke *et al*., 2013). Neighbour support typically involves help with practical errands, transport to services or social visits, social companionship, emotional guidance, and help with arranging professional care, and can therefore be understood as a form of informal care ( Broese van Groenou & De Boer, 2016). However, neighbour support also covers wider social arrangements. While acts of assistance can be one-way and directed towards those in need, neighbour support often blurs the boundary between care ‘giving’ and ‘taking’ and reflects wider moral emphases on reciprocity, mutual aid, self-help and ‘neighbourliness’ ( Boyce, 2006; Stephens *et al*., 2015; and Williams & Windebank, 2000). This points towards the key role of neighbour support in building place-based identity, sense of belonging, and ‘care for place’ ( Wiles & Jayasinha, 2013).

Evidence from a range of major global public health incidents shows that neighbour-based action plays a critical role in emergency response, assistance, resilience and recovery (e.g. Guo *et al*., 2020; Hawkins & Maurer, 2010; Jovita *et al*., 2019; Mayer, 2019; Noel *et al.*, 2018; and Sasaki *et al.*, 2020). Neighbour-based actions can be rapid, responsive and attentive to specific, locally felt needs. Small, spontaneous contributions at the street level can form a starting point for more organised actions, for example through place-based community development, and voluntary and civic action more broadly ( Talò *et al*., 2014). This reflects more general circumstances in which neighbour support can facilitate links between community members with social needs and formal care-givers, professionals and services ( Van Dijk *et al*., 2013).

There are, however, limitations and problems linked to neighbour support, both under ‘normal conditions’ and at times of major social upheaval. Assistance through these routes may be inconsistent and insufficient to address complex social needs. Neighbour support can amplify and effect existing social divisions in that it serves to reinforce existing patterns of privilege, or exclude disadvantaged groups from access to resources ( Ganapati, 2013; and Monteil *et al*., 2020). The responsibility for supporting neighbours often falls disproportionately on those already engaged in other forms of unpaid, obligated care, and can thereby replicate existing patterns of social division. Some actions obtain greater recognition, validation and prestige than others. These issues illustrate how the ideas of ‘neighbourhoods’ and ‘neighbour support’ differ according to time and place and how they are intimately shaped through power relations.

There is a large body of literature on neighbourhood social capital that explores the intersections between local geography, health and socio-economic inequalities ( Carpiano, 2006; and Moore & Kawachi, 2017). This body of literature explores both the potential psychosocial and material/structural impacts of social capital on health. There may be a virtuous circle of neighbour support in neighbourhoods that are already socially cohesive, integrated, and have high levels of personal and social assets. By contrast, certain forms of support in neighbourhoods affected by multiple deprivation may be constricted by access to wider material assets and networks, for example.

With regard to the coronavirus (COVID-19) pandemic and the associated lockdown restrictions, there is currently little available research on informal action at the neighbourhood level. In the UK, data on public behaviour indicate a substantial rise in informal and voluntary action at the neighbourhood level (e.g. Felici, 2020; and Tanner & Blagden, 2020). Notably, there appears to have been a surge in engagement with social media and online support groups since the pandemic started. In total, 22% of adults in the UK currently belong to some form of community support group, with more than a third of them joining since COVID-19 began to spread ( The Economist, 2020). It is estimated that about two million people in the UK have joined local support networks on Facebook, and the hyperlocal social network, Nextdoor, has seen a 90% rise in membership since the crisis began (ibid.). At a more formal level, a national call to register with the app-based NHS Volunteer Responders scheme led to over 750,000 applications ( NHS England, 2020). Such engagement is likely to be geographically uneven given socio-economic differences in the use of digital platforms ( Yates & Lockley, 2018).

Early evidence indicates that the coronavirus pandemic, and the wider governmental and societal response, disproportionately affects some social groups ( Bibby *et al.*, 2020; and The Guardian, 2020). Adjusting for age, COVID-19 deaths among people living in more deprived areas of England have been more than double those living in less deprived areas ( ONS, 2020). Beyond those directly at-risk of the virus, there are concerns about the wider impacts of COVID-19 and the measures taken to control the spread of the virus, including on people with mental health issues ( Carvalho *et al.*, 2020; and Holmes *et al*., 2020), isolated older people ( Armitage & Nellums, 2020), those in precarious employment ( Adams-Prassl *et al*., 2020), and women with care responsibilities ( Alon *et al.*, 2020), all of which intersect with area-based social inequalities ( Arcaya *et al*., 2015). It has also been suggested that local networks of support in response to the COVID-19 lockdown have predominated in areas of higher socio-economic status ( Felici, 2020).

We undertook this rapid research study – named ‘Apart but not Alone’ – given the potential significance of action at the neighbourhood level, and the limited opportunities to gather evidence as it is happening. Drawing upon the perspectives of people who feel they have been involved in supporting their neighbours in a UK urban setting, our main research question is: *What are the characteristics of neighbour support in the early stage of the COVID-19 lockdown, and how are these associated with area-based social deprivation?*

## Methods

### Design

This was a cross-sectional study to understand the key characteristics of neighbour support and associations with area-based deprivation.

### Study area

This study covered the ‘Bristol Built-Up Area’ as defined by the Office for National Statistics (ONS) with reference to the Bristol conurbation. This covers all the local government area of the city of Bristol, most of South Gloucestershire and an urban fringe of North Somerset. It is the eighth largest urban area in the UK with a population estimate of 746,049 in 2018 ( Centre for Cities, n.d.).

### Survey tool

The survey took place between weeks three and four of the UK Government’s Coronavirus Stay-at-Home Restrictions (COVID-19 lockdown), from 6 ^th^–20 ^th^ April 2020. We decided that a paper-based questionnaire would be difficult to send out through mailing lists due to office closures, and that the process would also constitute a risk for coronavirus transmission. We therefore developed a web-based questionnaire that combined structured and unstructured questions using Qualtrics, an online survey software. Questions covered what people thought of their neighbourhood, the types of neighbour support taking place, the groups of people supported, areas of success and challenge, and wider perceptions of personal involvement in the life of the neighbourhood. Respondents were asked to provide demographic information (gender, race/ethnicity, and age group) and postcode of current residence. The questionnaire was piloted and refined with the help of public contributors from People in Health West of England (PHWE) and Bristol Ageing Better (BAB) Community Researchers, both acting as a public involvement group for this study.

### Target population and recruitment process

The survey was intended to be completed by adults who self-identified as being involved in neighbour support, and was therefore not a survey of the general population. Respondents also, by default, were digitally-engaged through social media platforms such as Facebook, or through the mailing lists of voluntary groups. The online survey was publicised through several channels including nineteen postcode-based Facebook mutual aid groups, three coronavirus community and voluntary sector support groups for older people, two Black, Asian and Minority Ethnic (BAME) support groups, two disability support organisations, one social housing action group, and the local Healthwatch (an organisation representing public views on health and social care).

### Data analysis

All data were exported from Qualtrics to the SPSS Statistics software package version 25.0 ( IBM Corp., 2017) for further processing and analysis. Returned questionnaires were first screened to assess for 75% completion of questions and valid postcodes within the study area. The postcodes were matched to the ONS Local Super Output Area (LSOA) decile for the 2019 Index of Multiple Deprivation (IMD), a widely used official indicator for local area-based social deprivation ( MHCLG, 2019). A screening question filtered out respondents who were only engaged in supporting people who they did not see as their neighbours. The dataset was then analysed using frequencies and cross tabulations. Associations between the two levels of deprivation and each characteristic of neighbour support were tested for. The significance level was adjusted using the Bonferroni correction to account for multiple testing. Two members of the research team coded the text-based data following a thematic analytical approach ( Braun & Clarke, 2006).

### Ethical issues

Initial information about the study was provided in the online post/mail message. After clicking the survey link, respondents were provided with further information about the study, a statement on confidentiality, anonymity and the processing of data in accordance with the 2018 General Data Protection Regulation. They were then asked to give check box consent before proceeding to the questionnaire. Approval for the study was granted on 27 ^th^ March 2020 by the University of the West of England Health and Applied Sciences Research Ethics Committee, reference number HAS.16.11.045.

## Results

### Respondents

**Figure 1.**
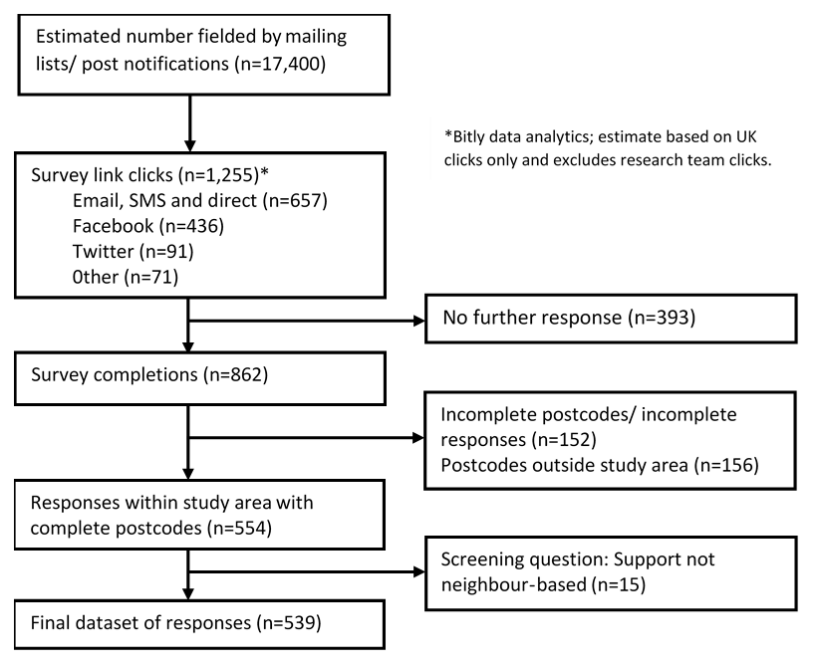
Flow chart of survey responses.

The process of recruiting survey respondents is illustrated in Figure 1. The link to the online survey was distributed to approximately 17,400 potential respondents, of whom 1,255 clicked on the link. Of these, 393 potential respondents who clicked on the survey link did not provide any further response. The number of potential respondents that completed and submitted the survey was 862, which equates to a completion rate of 68.7%. Of these submitted surveys, 308 were excluded either due to incomplete data (n=152) or because the postcodes provided did not meet our study area eligibility (n=156). A further 15 respondents were excluded based on non-neighbour support. The number of responses included for final analysis was 539.

### Socio-demographic profile of respondents

Table 1 shows that 80.9% (n=406) of survey respondents were female; 5.3% (n=26) of respondents were from BAME backgrounds. The age range of respondents were concentrated in middle adulthood, with 40–49 years as the modal age group (n=122). The postcode LSOA IMD decile shows that there were respondents from a range of areas, with more than a quarter of respondents (28.8%, n=155) from areas of lower deprivation (IMD Ranks 9 and 10).

**Table 1.** Proportion and number of survey respondents by socio-demographic characteristics.

Characteristics	Proportion (%)	Number
**Gender**		***Base = 502***
Female	80.9	406
Male	17.7	89
Prefer to self-describe	0.6	3
Prefer not to say	0.8	4
**Race/Ethnicity**		***Base = 490***
White (British, Irish, Other)	93.9	460
BAME	5.3	26
Prefer not to say	0.7	4
**Age range**		***Base = 494***
18–29 years old	7.5	37
30–39 years old	20.2	100
40–49 years old	24.7	122
50–59 years old	23.1	114
60–69 years old	18.0	89
70–79 years old	5.5	27
80 years or older	1.0	5
**Postcode LSOA IMD decile**		***Base = 539***
1 (Highest deprivation)	5.8	31
2	6.5	35
3	9.1	49
4	9.1	49
5	7.8	42
6	7.4	40
7	12.6	68
8	13.0	70
9	11.3	61
10 (Lowest deprivation)	17.4	94
**Vulnerable to/at risk of coronavirus** *		***Base = 496***
Respondent	16.3	81
Another person in the household	14.9	74
**Formal volunteering role** *		***Base = 493***
Registered as an NHS Volunteer Responder	10.1	50
Registered as a volunteer through the council	10.3	51
Local COVID-19 mutual aid/support group	7.3	36
Charity/NGO (general)	4.5	22
Food bank/ food charity	2.8	14
Faith group	2.0	10
Making supplies for the NHS	1.0	5
Other	4.7	23

*Responses are optional, therefore proportions do not add up to 100%.

A minority (16.3%, n=81) reported being at risk of or vulnerable to coronavirus, and 14.9% (n=74) reported that another person in their household was at risk.

A small proportion of respondents were engaged in some form of formal volunteering role with the NHS Volunteer Responders (10.1%, n=50), the council (10.3%, n=51), local COVID-19 mutual aid/support groups (7.3%, n=36), and a range of other groups such as charities, and faith-based organisations.

### Characteristics of neighbour support

The characteristics of neighbour support according to high (LSOA IMD Ranks 1–5; n= 206) and low (LSOA IMD Ranks 6–10; n=333) levels of area-based multiple deprivation are presented in Table 2.

**Table 2.** Differences in characteristics of neighbour support by area-based levels of multiple deprivation.

Characteristics	High IMD *n (%)*	Low IMD *n (%)*	Chi-square	df	*p-value*
**Routes of neighbour contact**					
Talking at a safe distance	173 (38.2)	280 (61.8)	0.018	1	0.893
Paper leaflets, newsletters or messages	69 (35.4)	126 (64.6)	1.124	1	0.289
Facebook	93 (40.3)	138 (59.7)	0.680	1	0.410
WhatsApp	139 (40.5)	204 (59.5)	2.005	1	0.157
Nextdoor	44 (35.2)	81 (64.8)	0.702	1	0.402
Email	42 (35.9)	75 (64.1)	0.392	1	0.531
Zoom/ FaceTime/ Skype	54 (35.5)	98 (64.5)	0.738	1	0.390
Text messages	104 (39.5)	159 (60.5)	0.311	1	0.577
Telephone calls	90 (39.0)	141 (61.0)	0.061	1	0.804
None of the above	0 (0.0)	1 (100)	0.624	1	0.430
Other	14 (45.7)	19 (54.3)	0.856	1	0.355
**Types of neighbour support offered**					
Collecting medical prescriptions for people isolating/ vulnerable	88 (36.8)	151 (63.2)	0.348	1	0.555
Doing food shopping for people isolating/ vulnerable	142 (35.9)	254 (64.1)	3.882	1	0.049
Sharing information and advice about coronavirus	110 (37.2)	186 (62.8)	0.304	1	0.582
Sharing information about food availability	126 (37.3)	212 (62.7)	0.336	1	0.562
Sharing tips for looking after children	53 (37.6)	88 (62.4)	0.029	1	0.866
Sharing humour/ lifting the mood	135 (36.7)	233 (63.3)	1.213	1	0.271
Sharing inspirational ideas	58 (37.9)	95 (62.1)	0.007	1	0.935
Walking dogs for other people	31 (35.6)	56 (64.4)	0.287	1	0.592
Window art, like rainbow pictures	100 (35.8)	179 (64.2)	1.404	1	0.236
Requests/ offers to give or lend items	112 (35.9)	200 (64.1)	1.739	1	0.187
Front garden tidy-ups/ beautifying	24 (46.2)	28 (53.8)	1.558	1	0.212
Identifying local helpers	72 (38.5)	115 (61.5)	0.013	1	0.909
Other	38 (36.5)	66 (63.5)	0.148	1	0.700
**Actions taken to reach out to neighbours who** **might need help**					
Putting leaflets through neighbour’s door	105 (37.5)	175 (62.5)	0.119	1	0.730
Contacting neighbours whose details you already have	101 (35.7)	182 (64.3)	1.645	1	0.200
Displaying messages on windows/ your property	33 (42.3)	45 (57.7)	0.664	1	0.415
Displaying messages on local noticeboards or in shops	13 (30.2)	30 (69.8)	1.255	1	0.263
Knocking on doors	20 (35.1)	37 (64.9)	0.259	1	0.611
**Area of neighbourhood support**					
Close neighbours, such as people next door	39 (44.8)	48 (55.2)	2.297	3	0.513
Neighbours in all or part of the street	69 (35.6)	125 (64.4)			
Neighbours on the street and surrounding streets	63 (37.3)	106 (62.7)			
Other: Larger area (Estate, district, ward etc.)	35 (39.3)	54 (60.7)			

The Stay-at-Home restrictions placed highly unusual constraints on the routes through which neighbours could contact one another. While face-to-face communication was the leading route (88%, n=453), respondents reported compliance to social distancing rules. It is worth noting that community-oriented social media platforms such as WhatsApp (66%, n=343) and Facebook (45%, n=231) featured in the responses, alongside well-established routes of communication such as phone calls (45%, n=231), text messages (51%, n=263), emails (23% n=117), next-door visits (24%, n=125), and paper-based messages such as leaflets and newsletters (39%, n=195). Digital platforms such as Zoom, FaceTime and Skype (29%, n=152) were also reported as means of maintaining contact with neighbours.

Respondents reported providing a wide range of support to their neighbours. Some of these involved direct support for people who were isolating - and unable to leave home - with food shopping and medical prescriptions (77%, n=239), support around food availability (66%, n=338), and providing information on coronavirus (57%, n=296). There was also broader support to raise the mood (71%, n=664), help inspire (30%, n=153), and otherwise improve the neighbourhood through creative actions (54%, n= 279). Few respondents reported helping to walk dogs (17%, n=87) and fewer still assisted with tidying others’ front gardens (10%, n=52).

Ahead of the lockdown, there was evidence that respondents were actively approaching their neighbours through existing contact details (55%, n=283), house-to-house leafleting (54%, n =280), and door-knocking (11%, n=57). A minority were also putting out messages on their property (15%, n=78) or other local places, such as local noticeboards and shops (8%, n=43).

Respondents were asked to choose an option that best described the size of the area in which their neighbours were supporting each other. Responses ranged from close neighbours, such as people next door (16.3%, n=117), to neighbours in all or part of the street (37.3%, n=194), the surrounding streets (31.7%, n=169) and a larger area (11.3%, n=89).

There were no statistically significant differences in any of the characteristics of neighbour support between areas of high and low levels of multiple deprivation (p> 0.002). However, for each characteristic of neighbour support, the proportion of responses trended towards being higher in the low deprivation group than the high deprivation group, which suggests higher overall prevalence of support activities in certain areas.

### Relationship between area-based multiple deprivation and neighbour support

Respondents living in areas of higher multiple deprivation were compared to those living in areas of lower multiple deprivation in terms of the focus of neighbour support ( Table 3). There were some clear priority groups regardless of deprivation level. These included ‘people self-isolating’ (83%, n=424) and people over 70-years-old (80%, n=409). Residence in areas of higher multiple deprivation was associated with support for the following groups that might be vulnerable to coronavirus and the effects of the lockdown: people experiencing financial issues [χ2 (1, *N* = 120) = 11.751, *p* = 0.001]; people with disabilities or reduced mobility [χ2 (1, *N*=231) = 11.309), *p* = 0.001]; people with an existing medical condition [χ2 (1, *N*=284) = 7.612, *p* = 0.006]; and people living in homes with no outdoor space [χ2 (1, *N*=74) = 9.018, *p* = 0.003].

**Table 3.** Associations between high and low levels of area-based multiple deprivation and neighbour support.

Neighbour support	High IMD, n	Low IMD, n	Chi-square	df	*p*-value	Odds ratio (95% CI)
Self-isolating	164	260	0.110	1	0.740	1.08 (0.67, 1.75)
Over 70-years-old	147	262	5.054	1	0.025 *	0.61 (0.39, 0.94)
Experiencing financial issues	62	58	11.751	1	0.001 **	2.05 (1.36, 3.10)
Disability or reduced mobility	107	124	11.309	1	0.001 **	1.85 (1.29, 2.66)
Existing medical condition	124	160	7.612	1	0.006 *	1.67 (1.16, 2.40)
Living alone	131	204	0.230	1	0.631	1.10 (0.75, 1.60)
Living in homes with no outdoor space	40	34	9.018	1	0.003 **	2.12 (1.29, 3.49)
Parents and children	81	118	0.760	1	0.383	1.18 (0.82, 1.69)
With non-childcare responsibilities	45	65	0.386	1	0.534	1.15 (0.75, 1.76)
NHS and social care workers	77	113	0.603	1	0.438	1.16 (0.80, 1.67)
Other key workers: education, transport, utilities, food industry etc.	53	74	0.815	1	0.367	1.21 (0.80, 1.82)

*p< 0.05

** p< 0.005

When adjusted for multiple comparison, the differences were only statistically significant for people experiencing financial issues, those with disabilities or reduced mobility, and people living in homes with no outdoor space. Respondents living in areas of higher multiple deprivation were twice as likely to be supporting people with financial issues as those living in areas of low multiple deprivation (OR = 2.05, 95%CI = 1.36, 3.10). In terms of supporting people with disabilities or reduced mobility and people living in homes with no outdoor space, the odds were 15% (OR = 1.85, 95%CI = 1.29, 2.66) and 112% (OR = 2.12, 95%CI = 1.29, 3.49) greater in areas of higher multiple deprivation compared to areas of lower multiple deprivation, respectively.

For people self-isolating, parents and children, people with care responsibilities other than children, NHS and social care workers, and other key workers in education, transport, utilities or food industry, there were no statistically significant associations between areas of higher and lower multiple deprivation in relation to neighbour support ( *p >* 0.002).

### Perceptions of official information and advice

**Figure 2.**
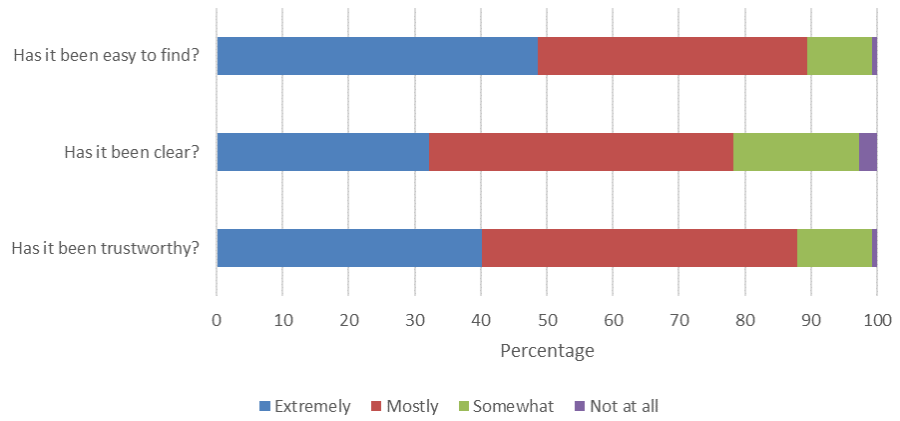
“From your point of view, how have you found the NHS/ Public Health England's information and advice on the coronavirus (COVID-19)?” (n=486).

Figure 2 indicates that there was a high level of satisfaction with official information and advice from the NHS and Public Health England. This was particularly the case for ease of access and trustworthiness, with a slightly weaker perception regarding the clarity of the information and advice.

Perception of official information about coronavirus did not differ between areas of higher and lower multiple deprivations. There were no statistically significant differences between higher and lower areas of multiple deprivation in relation to how easy it was to find official information about coronavirus, *t* (481) = 1.062, *p* = 0.289; the clarity of such information, *t* (480) = 1.661, *p =* 0.097; and the trustworthiness of the information, *t* (484) = 0.331, *p* = 0.741.

### Perceptions of the neighbourhood and involvement in neighbourhood life

Table 4 shows that nearly all respondents (97.4%, n=468) either agreed or strongly agreed with the statement “In my neighbourhood people are supporting each other very well at this time” However, there was a statistically significant difference between areas of high multiple deprivation (M = 1.61, SD = 0.54) and low multiple deprivation (M = 1.50, SD = 0.58) in terms of the responses to this statement ( *t* (478) = 1.980, *p* = 0.048).

**Table 4.** Responses to statements on perceptions of the neighbourhood and involvement in neighbourhood life.

Statement/ question	Response options	Proportion (%)	Number
**To what extent do you agree with the statement "In my neighbourhood,** **people are supporting each other very well at this time"? (n=486)**	*Strongly agree*	48.7	234
	*Agree*	48.7	234
	*Disagree*	2.6	9
**Please think back to before the coronavirus outbreak. How often** **would you say you took part in the life of your neighbourhood?** **(n=486)**	*More than most*	25.3	123
	*About the same*	38.3	186
	*Less than most*	26.3	128
	*Never took part*	10.1	49
**How do you think the coronavirus outbreak will affect your** **involvement in your neighbourhood in the future? (n=487)**	*I will want to get* *more involved*	46.2	225
	*I will continue the* *same as ever*	52.4	255
	*I will be less* *involved*	0.8	4
	*I don't want to get* *involved*	0.6	3

In terms of the extent to which respondents felt that they took part in the life of their neighbourhood before the coronavirus outbreak, it is notable that a total of 36.4% (n=177) stated that they took part either less than most or never took part. Looking to the future, there was a sign of intentions towards greater engagement overall, with 46.7% of respondents (n=225) stating that they wanted to get more involved in their neighbourhood.

However, perceptions of the neighbourhood and involvement in neighbourhood life did not differ between area-based multiple deprivation levels in relation to how they perceived their involvement in the life of their neighbourhood before the coronavirus outbreak, *t* (484) = 0.878, *p* = 0.380; and how they think the coronavirus outbreak will affect their involvement in their neighbourhood in the future, *t* (485) = 0.817, *p* = 0.414. Regardless of deprivation levels, of the 49 respondents who said they never took part in the life of their neighbourhood before the coronavirus outbreak, 38 (77.6%) wanted to get more involved in the future.

### Successes and challenges

Survey respondents were asked to give an open-text response to the statement *“Thinking about supporting people in your neighbourhood, please tell us about anything that has been successful or inspirational”.* Written statements covering a wide range of successes were provided by 372 respondents (69%). The main categories of successes are summarised in Table 5. Most of the successful accounts were categorised as small acts of kindness and developing social connections (n=114). Efficiency in communication through means such as social media and leafleting also featured prominently in the narrative descriptions of successes (n=105). The successful establishment of a local support group or the activities undertaken by an established group in response to the COVID-19 lockdown were also considered a positive development (n=82). One respondent captured a number of these elements in their description of both practical support and new connections which had been established through a social media platform post-lockdown:

**Table 5.** Key successes of neighbourhood support and their frequencies.

Key successes of neighbourhood support	Frequencies
Small acts of kindness and social bonding e.g. chats, assistance, friendly acts	114
Using communication routes successfully e.g. social media, leafleting	105
Setting up or operating a support group	82
Practical help, especially sourcing and delivering food or medicines	63
Creating community events e.g. social gatherings at a safe distance	62
Community spirit and solidarity (general)	37
Establishing/ re-establishing personal contacts	36
Acting quickly to address needs	34
Addressing sensitive issues, or reaching isolated/ vulnerable people	33
Voluntary action for wider society, beyond the neighbourhood	26
Sharing, swapping, or recycling	26
Positive actions of specific individuals	20
Supporting local businesses or services	15

“...A huge camaraderie and outpouring of generosity. Shared ‘shops’ have been a great help whilst self-isolating. We are introducing ourselves to each other with some family and historical background on WhatsApp. We plan to hold a street party at the end of all this” (ID: 145).

Survey respondents were also asked to give an open-text response to the statement “Thinking about supporting people in your neighbourhood, please tell us about anything that has been challenging or difficult”. This was answered by 324 respondents (60.1%). The main categories of challenges are summarised in Table 6. The major theme that emerged was difficulties in reaching out to people, including those perceived as most vulnerable (n=62). Other key challenges identified were those associated with social media communication in terms of its limited scope and other problems (n=43); inability to support others leading to frustrations (n=33) and developing trust with those who did not engage or were not willing to ask for help (n=32).

**Table 6.** Main challenges of neighbourhood support and their frequencies.

Main challenges of neighbourhood support	Frequencies
Challenges reaching most vulnerable or reaching out effectively	62
Limitations/ problems with social media communication	43
Frustration with not being able to help more	33
Establishing trust with those who do not engage or are unwilling to ask for help	32
Logistical challenges with food/medicine collection and delivery	30
Local tensions or anti-social behaviour	30
Issues with social distancing	29
Burden/risk-taking for some individuals	26
Poor community cohesion, social inequality and related effects	22
Tensions with organising activities	22
Stress/ risk with shopping and delivering food	17
Specific digital/ online problems	17
Stress, frustration or mental upset	17
Confidentiality, data protection or financial risks	16
Central or local government failings	13
Not following the government's rules	12
Sustaining support, getting fatigue or losing momentum	11

One respondent described their understanding of the challenge of reaching the most vulnerable in the context of neighbourhood support as follows:

“I personally think that the core function of providing support to the vulnerable can get swamped by the general effort to provide community support/morale (although very laudable and positive in its own right” (ID: 351).

Another described the difficulties faced establishing connection in their local area:

“Most of my neighbours keep to themselves. There is no sense of community. It’s difficult to help or be helped” (ID: 73).

Collectively, the logistical stress and virus contraction/transmission risk-taking aspects of sourcing and distributing food and medicine was also raised as a particular challenge (n=47).

## Discussion

This article reports on a survey of people engaged in supporting neighbours in the early stage of the COVID-19 lockdown in a major UK urban area. The findings show that survey respondents used a wide variety of approaches to communicate with neighbours and made greater efforts to contact vulnerable groups. Neighbours undertook tasks directly linked to the effects of the lockdown, such as obtaining and delivering food and medical prescriptions, as well as a variety of actions related to informing, advising and the sharing of experiences. A substantial proportion felt that they had become more involved in neighbourhood life following the lockdown and had an interest in becoming more involved in future. Neighbour support spanned all adult age groups, including older people categorised as being at risk of contracting the virus. With respect to most measures, there were no differences in the characteristics of support between respondents in areas of higher and lower deprivation. However, respondents from more deprived areas were more likely to state that they were involved in supporting certain vulnerable groups, and less likely to strongly agree that neighbours were supporting each other well.

### The support was quick, flexible and responsive

In the majority of cases, neighbour support appears to have been mobilised prior to the government Stay-at-Home restrictions. The focus of the support ranged from meeting essential personal needs (food and medicine) to broader social assistance and mutual aid and morale-boosting humorous and creative exchanges. The wide range of activities reported highlights the informality of the social connections, and the scope for a plural and multi-directional web of gifting and reciprocity. This neighbour support was, therefore, more expansive than the services available through formal voluntary channels such as the NHS Volunteer Responders scheme. Drawing upon the dates of the survey, this neighbour support was likely to have been organised more rapidly than that available through public and voluntary sector organisations, and was potentially able to adapt to changing demands. Unlike formal volunteering, there are limited opportunities to assess the quality, consistency and inclusivity of support given by neighbours.

The findings, however, also reveal that the informal and formal intersected, at least for a minority of respondents that reported being involved both in supporting their neighbour and engaging with voluntary organisations. In the COVID-19 crisis, it is plausible that such individuals were well-placed to transfer intelligence and best practice between neighbourhood and greater social scales of voluntarism.

### Social media platforms offer new possibilities for hyperlocal networking

The COVID-19 lockdown has highlighted the scope for setting up hyperlocal communities using social media platforms, such as WhatsApp and Nextdoor. A majority of survey respondents made use of such channels, enabling them to become involved in extended networks, which met a variety of neighbourhood needs. It was evident that this is a digital space with rapidly evolving potential. Clearly, the platforms opened alternative routes for communication, given the restrictions on physical proximity as a result of the pandemic. They also provide access to a way to legally share personal details, wider digital resources and, potentially, help disseminate official advice ( Guo *et al.*, 2020), and our findings indicate that these platforms played a key role in the exchange of key information. Our study supports other indications that social media has had a key place in the lives of many community activists ( Leong *et al.,* 2019). However, the full benefits and drawbacks of social media as part of crisis response, transition and recovery remain unclear and require further research.

### Geographical inequalities in neighbour support, and other potential disparities

There was evidence of differences in the type of support provided, based on the social geographies of neighbourhoods. In areas of higher deprivation, respondents were more likely to be addressing the needs of people with disabilities or reduced mobility, and people living in homes with no outdoor space. Respondents living in areas of higher multiple deprivation were twice as likely to report supporting people with financial issues as their counterparts in less deprived areas. This echoes other studies on area-based inequalities, social capital, and community responses to emergencies and disasters ( Hawkins & Maurer, 2010).

While there were many similarities in all areas between routes and types of actions for neighbour support, the evidence showed that respondents in areas of higher multiple deprivation were less likely to strongly agree that they and their neighbours were supporting each other well. This finding is consistent with much previous research on the relationships between neighbourhood social capital, health and wellbeing. Nevertheless, some caution is needed in this interpretation. We were not able to ask why people in lower income communities felt this way. Furthermore, some definitions of social capital have been criticised for ignoring the importance of social networks which are not able to mobilise significant resources, but which are nevertheless important in community Life ( Defilippis, 2001)

ONS official measure of area-based deprivation is a widely used indicator of the personal circumstances of residents and can be mapped across as an indicator of neighbourhood social capital ( Verhaeghe & Tampubolon, 2012). However, individuals in the same locality live in heterogeneous circumstances and neighbourhood social capital is not synonymous with individual social capital ( Waverijn *et al*., 2017). Furthermore, survey-derived methods tend to focus mostly on forms of bonding social capital – where deprived areas score lower as they have less homogenous relationships and are therefore, less inward-looking – and exploring bridging and linking social capital can change how that relationship is seen ( Poortinga, 2012).

Further aspects of our study point towards other potential forms of inequity in the context of the coronavirus pandemic which may require further examination. The challenges identified in reaching the most vulnerable, and the limitations and problems faced in employing social media raise issues of unequal communication and outreach. Although targeted invitations to complete the survey were made through the email support network mailing lists of older people and BAME groups, the proportions of response to the survey were low from these groups. While this in part reflects a study limitation (see below), it may indicate lower engagement with social media-driven neighbourhood support groups, for example. The low proportion of responses from males may, similarly, reflect higher levels of female engagement in such support provision. Indeed, this would be consistent with wider research on gender-based inequalities in informal care ( Pinquart & Sörensen, 2006) as well as disproportionate responsibilities for practical and emotional in the domestic and familial spheres under lockdown.

### Neighbour-based events as a basis for future action

Our study showed that the coronavirus pandemic and associated lockdown measures led to individuals taking diverse action to support their neighbours. This mobilisation has involved a mix of those individuals who were themselves as already involved in their local community broadening their scope of activity, as well as those who took this on as felt that this was a new activity. While our study did not measure people’s motivations, their accounts of their activities and reported successes and challenges indicate that the experience was often meaningful and rewarding. The COVID-19 crisis has led to new and revived local organisational structures – such as mutual aid groups – and has extended social networks, potentially also making them more diverse. Although evidence from other mass social events, such as the 2012 London Olympics, suggest that much of this voluntary action will be temporary ( Koutrou *et al*., 2016), the initial phase of the lockdown indicates a basis for reconstituting neighbourhood life through the recovery phase and beyond. Some of this potential was evidenced in the high proportion of survey respondents stating that they wished to become more involved in their neighbourhood in the future. Certain challenges identified are worthy of further research and we are conducting qualitative research and a second, follow-up survey to explore in more detail how neighbourhood responses have evolved over the extended lockdown period and what this might mean for future support initiatives.

### Strengths and limitations of the study

This study reports on a rapid survey in the context of a fast-moving, unanticipated and unprecedented viral pandemic. The data capture tools were guided by prior relevant research, piloted with the aid of public involvement, and implemented eight days after ethical approval. The survey was disseminated through a range of digital channels to local area groups and diverse communities of interest. The responses indicate that a range of people identified with the term ‘neighbour support’ and wished to report their experiences. Given limited options for contact, the survey results were from people with digital access, who self-identified with and wished to self-report neighbour support. Although we were able to control for some socio-demographic variables, the cross-sectional design does not allow us to fully interpret the meaning of lower responses for some categories. For example, the results show that 5.3% of respondents were from BAME backgrounds, compared to an estimated 15% for the study area.

### Implications and future research

This study points towards a number of issues for further work. Research is needed to more fully understand the intersections between informal neighbour support and formal voluntary and professionally-led action in the context of the coronavirus pandemic. Such enquiry has a role in informing effective responses to similar crisis situations in the future. The survey data points towards complex neighbour-based relationships that would benefit from qualitative research, particularly in the context of hyperlocal communication technologies. Further research is also needed to inform how crisis events, such as a major viral pandemic, can stimulate a legacy of inclusive civic action across issues that concern place-based communities.

## Conclusion

To our knowledge, ‘Apart but not Alone’ is the first academic research study of the characteristics of neighbour support during the COVID-19 lockdown. We provide evidence that there have been wide-ranging actions at the level of small urban areas and that these are likely to have been rapid, responsive and closely aligned to locally-felt needs. Therefore, neighbour support constitutes a notable field of informal social action that is likely to both complement and enhance the actions of formal agencies, and form an important basis for transition and recovery from the pandemic. While some aspects of neighbour support are similar in different locales, our study indicates that there are area-based disparities that are likely to increase over time in relation to existing experiences of deprivation.

## Data availability

### Underlying data

Figshare: Apart but Not Alone: survey 1 research data April 2020. Neighbour support and the covid-19 lockdown, https://doi.org/10.6084/m9.figshare.12301172.v3 ( Jones *et al*., 2020).

### Extended data

Figshare: Apart but Not Alone: survey 1 research data April 2020. Neighbour support and the covid-19 lockdown, https://doi.org/10.6084/m9.figshare.12301172.v3 ( Jones *et al*., 2020).

This project contains the following extended data:

- Questionnaire

Data are available under the terms of the Creative Commons Attribution 4.0 International license (CC-BY 4.0).
