# Supplementary material for: Apart but not Alone? A cross-sectional study of neighbour support in a major UK urban area during the COVID-19 lockdown
Source: Emerald Open Res. 2020 Jun 10;2:37. doi: 10.35241/emeraldopenres.13731.1 (PMC7390607; doi:10.35241/emeraldopenres.13731.1)
Supplement: Peer review history

# Peer review: Reviewer response for version 1

**DOI:** 10.21956/emeraldopenres.14806.r26971

**Published:** 2020-07-28

**Authors:** Aleksandra Kazlowska

**Affiliations:** 1 Department of Social Medicine and Public Health, Warsaw Medical University, Warsaw, Poland

**Basic reporting**

 The article “Apart but not Alone? A cross-sectional study of neighbour support in a major UK urban area during the COVID-19 lockdown” consists of abstract, introduction, methods, results, discussion and references, which include 45 items. In all parts, Authors provide the acceptable format of ‘standard sections’. The article is written in clear and unambiguous English. Tables are relevant to the content of the article and briefly summarize the main results. The article include a sufficient introduction and background to demonstrate that there is no data on informal action at the neighbourhood level during the coronavirus (COVID-19) pandemic and the associated lockdown restrictions.

**Experimental design**

 Authors clearly define the research question which was to find how people were engaged in supporting their neighbours in weeks three and four of the UK COVID-19 lockdown. This study allows to suggest that responses to the viral pandemic and associated social restrictions may increase existing social and health inequalities.  It is important that authors used comprehensive survey. Authors have described methods with sufficient information to be reproducible by another investigator.

**Validity of the findings**

 The analysis of data is beneficial and clearly stated. Interestingly, responders felt that they had become more involved in neighbourhood life following the lockdown and had an interest in becoming more involved in future.

 I commend the authors for their extensive data set. If there is a weakness of the study,  it is seen in a data analysis. A majority of responders were female. It is an important fact, that should be more highlighted. The result is more concentrated on  female’s neighbour support in a major UK urban area during the COVID-19 lockdown. The sample size of participants is impressive what is making your analyses statistically sound, and controlled and acceptable in a discipline. Conclusions are well stated, linked to an original research question.

**General comments for the author**

 In my opinion, the manuscript should be published in Emerald Open Research.

Is the work clearly and accurately presented and does it cite the current literature?

Yes

If applicable, is the statistical analysis and its interpretation appropriate?

Yes

Are all the source data underlying the results available to ensure full reproducibility?

Yes

Is the study design appropriate and is the work technically sound?

Yes

Are the conclusions drawn adequately supported by the results?

Yes

Are sufficient details of methods and analysis provided to allow replication by others?

Yes

Reviewer Expertise:

public health, human diet, pregnant

I confirm that I have read this submission and believe that I have an appropriate level of expertise to confirm that it is of an acceptable scientific standard.

# Response

**Published:** 2020-07-28

**Authors:** Mat Jones

**Affiliations:** University of the West of England, UK

Dear Aleksandra, Thank you very much for your positive review. This issue of low male responses is an interesting point that would certainly be of interest to investigate in similar research on public health crises and neighbour support.

 My regards

 Mat

# Peer review: Reviewer response for version 1

**DOI:** 10.21956/emeraldopenres.14806.r26885

**Published:** 2020-07-20

**Authors:** Niki Koutrou

**Affiliations:** 1 School of Sport and Exercise Sciences, University of Kent, Canterbury, UK

The topic of this paper is very interesting, contemporary and complex one, addressing the impacts of COVID-19 in raising community spirit, activism and social capital at neighbourhood level through informal, non-affiliated actions. There is very limited research to date addressing this issue, in particular in relation to COVID-19. The review draws on contemporary and relevant literature. The methodological underpinnings adopted are appropriate, justified and reproducible. The sample size is appropriate. The findings emerged through appropriate and rigorous statistical techniques and thus enhance our understanding of the drivers and manifestations of community support in times of public health emergencies and illustrate the differences between residents in areas of high and low deprivation. The conclusions are sound, and make appropriate links to relevant literature. On these grounds, I recommend, it is accepted for publication.

Is the work clearly and accurately presented and does it cite the current literature?

Yes

If applicable, is the statistical analysis and its interpretation appropriate?

Yes

Are all the source data underlying the results available to ensure full reproducibility?

Yes

Is the study design appropriate and is the work technically sound?

Yes

Are the conclusions drawn adequately supported by the results?

Yes

Are sufficient details of methods and analysis provided to allow replication by others?

Yes

Reviewer Expertise:

community volunteering, volunteer legacy, community action, social capital, mixed-methodology, statistical analysis

I confirm that I have read this submission and believe that I have an appropriate level of expertise to confirm that it is of an acceptable scientific standard.

# Response

**Published:** 2020-07-23

**Authors:** Mat Jones

**Affiliations:** University of the West of England, UK

Dear Niki,

 Thank you for your very positive review. We really appreciate that you have highlighted the topicality and wider significance of the study.

 Regards

 Mat and the team
